# Hippo Pathway Phylogenetics Predicts Monoubiquitylation of Salvador and Merlin/Nf2

**DOI:** 10.1371/journal.pone.0051599

**Published:** 2012-12-14

**Authors:** Robert G. Wisotzkey, Charlotte E. Konikoff, Stuart J. Newfeld

**Affiliations:** 1 NextBio, Santa Clara, California, United States of America; 2 Department of Biology, University of Washington, Seattle, Washington, United States of America; 3 School of Life Sciences, Arizona State University, Tempe, Arizona, United States of America; Rush University Medical Center, United States of America

## Abstract

Recently we employed phylogenetics to predict that the cellular interpretation of TGF-β signals is modulated by monoubiquitylation cycles affecting the Smad4 signal transducer/tumor suppressor. This prediction was subsequently validated by experiments in flies, frogs and mammalian cells. Here we apply a phylogenetic approach to the Hippo pathway and predict that two of its signal transducers, Salvador and Merlin/Nf2 (also a tumor suppressor) are regulated by monoubiquitylation. This regulatory mechanism does not lead to protein degradation but instead serves as a highly efficient “off/on” switch when the protein is subsequently deubiquitylated. Overall, our study shows that the creative application of phylogenetics can predict new roles for pathway components and new mechanisms for regulating intercellular signaling pathways.

## Introduction

Phylogenetic analyses of amino acid conservation in developmental signaling pathways have traditionally focused on pathway origins [1] or evolutionary relationships within one/two families that encode pathway components [2]. Recently we stepped outside the box and applied phylogenetics to questions of pathway regulation. Our analyses of the TGF-β pathway predicted regulation by novel phosphorylation events and monoubiquitylation affecting Smad signal transducers [3], [4]. Monoubiquitylation does not lead to protein degradation but instead serves as a highly efficient “off/on” switch when the protein is subsequently deubiquitylated [5]. Both of these predictions were validated by experiments in flies, frogs and mammalian cells demonstrating that the predicted regulatory mechanisms are fundamental features of TGF-ß signaling [6],[7],[8],[9].

**Figure 1:**
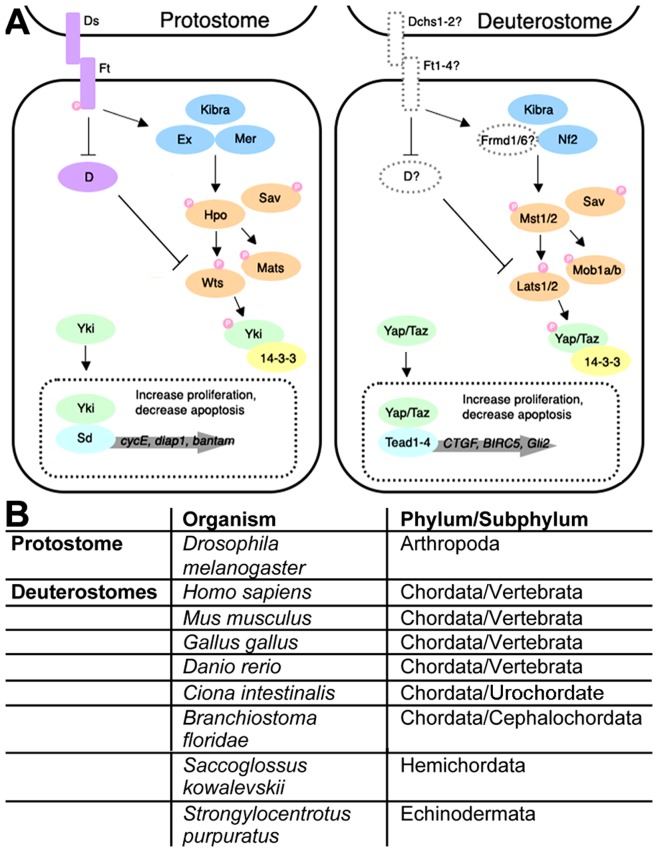
Schematic of the Hippo kinase pathway in flies and humans. A) Left side. In the protostome *D. melanogaster*, the transmembrane atypical cadherin ligand Dachsous (Ds) activates the transmembrane atypical cadherin receptor Fat (Ft). Fat then inhibits Dachs (D) and activates Expanded (Ex). Inhibition of Dachs prevents it from destabilizing the Warts (Wts) serine-threonine kinase and activation of Expanded leads to increased kinase activity of Warts. Expanded accomplishes this via a complex containing Merlin and Kibra that facilitates phosphorylation of the Hippo (Hpo) serine-threonine kinase, the Warts kinase and their respective co-factors Salvador (Sav) and Mats. Warts then phosphorylates the transcription co-activator Yorkie (Yki). Phosphorylated Yorkie is bound by 14-3-3 proteins and sequestered in the cytoplasm. When not phosphorylated, Yorkie translocates to the nucleus, binds transcription factors such as Scalloped (Sd), and influences target gene expression leading to increased cell proliferation and decreased apoptosis. A subset of known target genes is shown. Right side. In the deuterostome *H. sapiens,* proteins are shown in the same color and subcellular location as their corresponding *D. melanogaster* proteins: Hippo is Mst1/2, Warts is Lats1/2, Mats is Mob1a/b, Yorkie is Yap/Taz, Scalloped is Tead1/2/3/4, Expanded is Frmd1/6, and Merlin is Nf2. The roles of mammalian homologs of Dachsous, Fat, Expanded and Dachs have not yet been confirmed and they are shown with dashed lines and no color. Mammalian target genes are not homologs of the fly target genes. B) Formal names of the nine coelomate species in this study and their phylum/subphylum classifications.

Here we report a phylogenetic analysis of nine families that participate in the Hippo pathway and compare the results to the TGF-β (five families) and Wnt (ten families) pathways [10],[11]. Our goal is to predict new regulatory mechanisms. Hippo pathway activation in flies (Figure 1A left) begins when a signal from a neighboring cell is communicated through two atypical transmembrane cadherins - the ligand Dachsous and its receptor Fat. Fat then initiates two cascades that converge synergistically downstream. Fat blocks the activity of the myosin-like protein Dachs by preventing it from accumulating at the membrane. Inhibition of Dachs leads to increased stability of the Warts serine-threonine kinase. Fat also increases the membrane association of the Ferm domain protein Expanded that leads to an increase in the activity of Warts. Expanded accomplishes this via a complex containing the Ferm domain protein Merlin and the WW domain protein Kibra. This complex then recruits the Hippo kinase complex (Hippo, Salvador, Warts and Mats) to the membrane where Hippo and Salvador undergo phosphorylation via an unknown mechanism.

The Hippo serine-threonine kinase, assisted by its co-factor Salvador, then phosphorylates Warts and its co-factor Mats. Salvador and Mats are WW/Sarah and Phoecin domain adapter proteins, respectively. Phosphorylated Warts then phosphorylates Yorkie, a WW domain transcription co-factor. Phosphorylated Yorkie is recognized and sequestered in the cytoplasm by 14-3-3 proteins. In the absence of phosphorylation Yorkie continually translocates to the nucleus, binds the transcription factor Scalloped and influences gene expression that leads to increases in cell proliferation and decreased apoptosis [12]. In vertebrates (Figure 1A right), Hippo signaling is similar and elicits similar outcomes, but not all homologs of fly Hippo pathway genes have been implicated in the vertebrate Hippo pathway [13].

Previously, a phylogenetic study of the Yorkie/Yap/Taz family and its binding partners in the Scalloped/Tead family revealed evidence of co-evolution [2]. Another study focused on pathway origins showed that the Hippo/Mst, Warts/Lats, Yorkie/Yap/Taz and Scalloped/Tead families are present in a unicellular amoeboid species and that the amoeboid proteins can promote tissue growth in *Drosophila* [1]. The phylogenetics data we present have a larger scope then either of these studies: we analyze nine Hippo pathway families and compare the results to data for TGF-ß and Wnt pathway families.

Three lines of phylogenetic data converge to support predictions that two non-enzymatic Hippo pathway signal transducers, Salvador and Merlin/Nf2 are strong candidates for regulation by monoubiquitylation. Merlin/Nf2 is a well-known tumor suppressor and there is suggestive experimental evidence in the literature consistent with our prediction. From a larger perspective, our study demonstrates that the creative exploitation of amino acid sequence conservation via phylogenetics can predict new roles for pathway components and new regulatory mechanisms influencing developmental signaling pathways.

## Results and Discussion

### Experimental Design for Phylogenetic Predictions of Lysine Monoubiquitylation

Phylogenetic analyses of amino acid alignments have successfully improved our understanding of TGF-β pathway regulation. The underlying logic of the phylogenetic approach is to exploit evolutionary conservation as a guide to amino acids involved in the post-translational regulation of protein activity. As both mono- and polyubiquitylation occur solely on lysine (with very rare exceptions) the identification of potential monoubiquitylation sites requires consulting available biochemical data to eliminate lysines conserved for structural reasons. Monoubiquitylation predictions for a specific lysine are then based on: 1) universal conservation in a broad range of species, 2) occurrence within an highly conserved context and 3) that is not essential for protein structure [4].

In this study we also required that the conserved context include an upstream hydrophobic residue (at positions -1 or -2) as we noted for the two monoubiquitylated lysines (K519 and K507) in human Smad4 [8],[14]. The possibility that an upstream hydrophobic residue is part of a monoubiquitylation “signature” is supported by several computational analyses. These showed that the context is a significant factor in ubiquitylation [15], that hydropathy is the most important feature of the context for ubiquitylation [16] and that polyubiquitylation is associated with a statistically significant depletion of the hydrophobic residue leucine at positions -1 and -2 [17]. Thus the presence of a hydrophobic amino acid in these positions may allow the ubiquitin ligase to discriminate between poly- and monoubiquitylation.

The nine highly divergent species employed in this study and their Linnaean classifications are shown in Figure 1B. All are coelomates with three embryonic germ layers and a digestive tract with two openings: eight are deuterostomes (blastopore becomes the anus) and one is a protostome (blastopore becomes the mouth). Deuterostomes and protostomes diverged 1.1 billion years ago. Within the deuterostomes, echinoderms (sea urchins) and hemichordates (acorn worms) diverged 990 and 900 million years ago (mya) from chordates. Within the chordates, cephalochordates (lancelets) and urochordates (ascidians) diverged 750 and 720 mya from vertebrates. Within the vertebrates, zebrafish diverged from amniotes 460 mya. Within the amniotes, chickens diverged from mammals 330 mya. Within mammals, mice diverged from humans 93 mya [18].

### Salvador and Merlin/Nf2 Lysine Conservation Resembles Smad4 Lysine Conservation

In the Hippo family, human Mst1 contains thirty-six lysines (Table 1). Eighteen (50%) of these are universally conserved but biochemical data suggests that fifteen are structural [19]. We examined these fifteen lysine residues according to the criteria noted above (universal conservation, conserved context, upstream hydrophobic residue and not structural) and estimated a qualitative likelihood of monoubiquitylation. Based on a hydrophobic leucine at position -1 in all species and its location upstream of the kinase domain we predict that Mst1 K15 is a modest candidate for monoubiquitylation.

**Table 1:** Summary of absolutely conserved lysine residues and predictions for monoubiquitylation candidates in human Hippo pathway proteins.

Protein Family	Conservedlysines	Total lysines	Conservedfraction	Monoubiquitylation candidate lysine^a^	Conservedcontext^b^
Hippo/Mst	18Hs_Mst1	36	50%	K15 just upstream of the kinase domain is a modest candidate	**L-** K-K-L-S/Din all species
Salvador	1Hs_Sav1	18	5.6%	K345 in the Hippo/Mst1 binding domain is a strong candidate	**M**-K-E-L-Ein vertebrates
Warts/Lats	14Hs_Lats1	64	21.9%	K652 in the Yap/Taz binding domain is astrong candidate	**I/L/M**-K-S/Tin all species
Mats/Mob	8Hs_Mob1a	17	47.1%	K104 just upstream of the dimerizationdomain is a strong candidate	**I/V**-K-K-P-Iin all species
Yorkie/Yap/Taz	0Hs_Yap1	14	0%	K90 in the Scalloped/Tead bindingdomain is a modest candidate	**L**-R-K-Lin vertebrates
Scalloped/Tead	12Hs_Tead1	28	42.9%	K289 in the Yap/Taz binding domain is astrong candidate	**L**-**V**-K-F-W-Ain all species
Kibra/Wwc	1 Hs_Wwc1	74	1.4%	K43 between two Wwc binding domains is a modest candidate	**L**-T-K-Pin vertebrates
Expanded/Frmd	7Hs_Frmd1	20	35%	K116 in the first Ferm binding domain is astrong candidate	**I/L**-S/Y-K-Yin all species
Merlin/Nf2	7Hs_Nf2	60	11.7%	K322 between lipid -binding and ERMdomains is a strong candidate	**M-** K-A-Q-A-Rin all species

a If only one conserved lysine is present, then we consider it a monoubiquitylation candidate. If multiple conserved lysines are present, then we identify a candidate for monoubiquitylation (underlined). Qualitative assessment of the likelihood that the lysine is monoubiquitylated is indicated (strong or modest).

b Absolutely conserved amino acids surrounding the candidate lysine (underlined) are shown. If amino acids are separated by a slash then that position is variable and the amino acids present are shown. A hydrophobic amino acid (I, L, V or M) in the -1 or -2 position is shown in **bold**.

Human Salvador1 contains eighteen lysines (Table 1) of which only one (5.6%) is universally conserved. Salvador K345 occurs within the Sarah domain that binds Hippo/Mst1 but biochemical data suggests it is not structural [20]. K345 monoubiquitylation could block Salvador/Hippo complex formation just as Smad4 monoubiquitylation at K519 blocks Smad4/Smad2 binding [8]. In addition, Salvador K345 has a hydrophobic methionine at -1 in vertebrates and a leucine at -2 in all other species. The similarity between Smad4 K519 and Salvador K345 led us to predict that K345 is a strong candidate for monoubiquitylation.

Differences in lysine conservation between Salvador and the polyubiquitylated Armadillo/ß-catenin family are consistent with the prediction of Salvador K345 monoubiquitylation. Human ß-catenin contains twenty-six lysines of which three (11.5%) are universally conserved. These lysines are structural components of the interaction surface that binds the transcription factor TCF [21]. Further, in contrast to the monoubiquitylated K519 of Smad4 that is conserved in *C. elegans* as sma-4 K538, the polyubiquitylated K19 in human ß-catenin is not conserved in *C. elegans* wrm-1 [10]. Since polyubiquitylation need not occur within an interaction domain, the target lysine is not always conserved.

In the Warts family, human Lats1 contains sixty-four lysines (Table 1) of which fourteen (21.9%) are universally conserved. Based on the structure of the related Akt serine-threonine kinase, ten of these appear structural [22]. Lats1 K652 occurs in the Yorkie/Yap/Taz interaction domain, has a hydrophobic residue at -1 in all species and is predicted to be ubiquitylated by the UbiPred algorithm [17]. We consider K652 a strong monoubiquitylation candidate. In the Mats family, human Mob1a contains seventeen lysines (Table 1) of which eight (47.1%) are universally conserved and three are structural [23]. Mob1a K104 is located upstream of the dimerization interface, has a hydrophobic residue at -1 in all species and is predicted by UbiPred. We consider K104a strong monoubiquitylation candidate.

In the Yorkie family, human Yap1 has fourteen lysines (Table 1) of which none (0%) are universally conserved. The absence of lysine conservation is unparalleled among the twenty-four families of the Hippo, TGF-ß and Wnt pathways we have studied [4],[10]. Two human Yap1 lysines (K90 and K204) are conserved within vertebrates and since K90 has a leucine at -1 and K204 is predicted by UbiPred, we consider these modest monoubiquitylation candidates. Consistent with the ß-catenin data, K97 and K242 that were shown to be polyubiquitylated in mouse Yap1 [24],[25] are not conserved outside mammals. In the Scalloped family, human Tead1 has twenty-eight lysines (Table 1) of which twelve (42.9%) are universally conserved and six are structural [26]. Tead1 K289 is located in the Yorkie/Yap/Taz interacting region, has a leucine at -2 and a valine at -1 in all species and is predicted by UbiPred. We consider Tead1 K289 a strong monoubiquitylation candidate.

In the Yorkie family, human Yap1 has fourteen lysines (Table 1) of which none (0%) are universally conserved. The absence of lysine conservation is unparalleled among the twenty-four families of the Hippo, TGF-ß and Wnt pathways we have studied [4],[10]. Two human Yap1 lysines (K90 and K204) are conserved within vertebrates and since K90 has a leucine at -1 and K204 is predicted by UbiPred, we consider these modest monoubiquitylation candidates. Consistent with the ß-catenin data, K97 and K242 that were shown to be polyubiquitylated in mouse Yap1 [24 25] are not conserved outside mammals. In the Scalloped family, human Tead1 has twenty-eight lysines (Table 1) of which twelve (42.9%) are universally conserved and six are structural [26]. Tead1 K289 is located in the Yorkie/Yap/Taz interacting region, has a leucine at -2 and a valine at -1 in all species and is predicted by UbiPred. We consider Tead1 K289 a strong monoubiquitylation candidate.

In the Kibra family, human Wwc1 has seventy-four lysines (Table 1) of which only one (1.4%) is universally conserved. Wwc1 K43 has a leucine at -2 in vertebrates and is located within a WW binding domain. As no structural data on human Wwc1 is available, we consider K43 a modest candidate for monoubiquitylation in vertebrates. In the Expanded family, human Frmd1 has twenty lysines (Table 1) of which seven (35%) are universally conserved and two are structural [27],[28]. Frmd1 K116 has a hydrophobic residue at -2 in all species, is located within a Ferm interaction domain and is predicted by UbiPred. We consider K116 a strong candidate for monoubiquitylation.

In the Merlin family, human Nf2 has sixty lysines (Table 1) with seven (11.7%) universally conserved and three structural [27],[28]. K322 has a conserved hydrophobic residue at -1, is located between lipid binding and ERM domains and is predicted by UbiPred. We consider K322 a strong candidate for monoubiquitylation. After making this prediction, we found evidence in the literature suggesting that K322 is monoubiquitylated [29]. In that paper, an N-terminal fragment containing residues 1–322 of human Nf2 was monoubiquitylated in cell culture by an endogenous ligase (as shown in that paper’s Figure 4E upper right panel lane 2). Upon replacing a serine or threonine with a phosphomimetic glutamic acid (T230D and/or S315D) this fragment became polyubiquitylated (as shown in that paper’s Figure 4E upper right panel lanes 3,4). However, none of the targeted lysines were identified. That paper’s results strongly support our prediction because monoubiquitylation, as noted above, favors conserved lysines and K322 is the most upstream of the seven conserved lysines in Merlin/Nf2 (there is no other conserved lysine within 1–322) and polyubiquitylation favors unconserved lysines (there are twenty-five unconserved lysines within 1–322). Note that Merlin/Nf2 has roles in cell fate specification that are not connected to canonical Hippo signaling [30] and this prediction does not allow us to specify which of Merlin/Nf2’s functions may be regulated by monoubiquitylation.

In summary, the lysine conservation data most strongly predicts the monoubiquitylation of Salvador and Merlin/Nf2. For Salvador, this is based on the presence of only one conserved lysine that is not required structurally and that is present in a conserved context containing a hydrophobic residue at -1 or -2 in all species. For Merlin/Nf2, where suggestive experimental data exists in the literature, this is based on the presence of a conserved lysine that is not required structurally and that is present in a conserved context containing a hydrophobic residue at -1 in all species.

### Salvador and Merlin/Nf2 Copy Number Reduction in Mammals Mimics Smad4 Copy Number Reduction

We then sought to identify additional parallels between Hippo pathway families and monoubiquitylated members of the Co-Smad subfamily (Smad4 in vertebrates and Medea in flies). A survey showed that vertebrate species in all Hippo families contain a variable number of family members.

**Figure 2:**
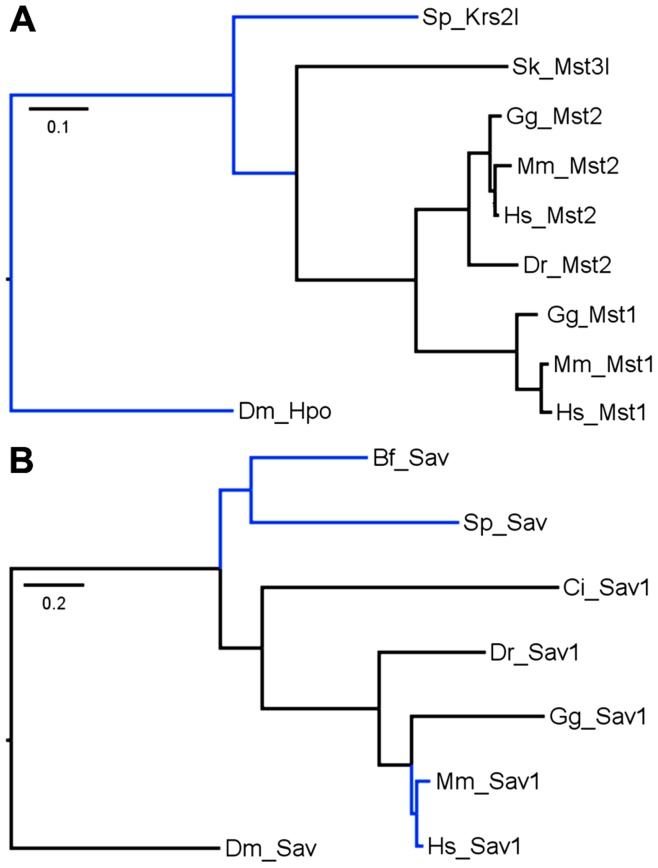
Hippo/Mst and Salvador Maximum Likelihood trees. Trees rooted with *D. melanogaster* are shown. Branches considered statistically weak (aLRT values <0.70) are colored in blue. Branch lengths are drawn to scale and correspond to the average number of amino acid changes per site as indicated by the scale bar. A) Hippo/Mst vertebrate topology matches the species tree. Among invertebrates the hemichordate strongly clusters with vertebrates while the echinoderm is an outlier. The overall Hippo/Mst tree matches the species tree. The Bayesian tree (Figure S1A) shows one difference - the hemichordate moves out of the strong cluster with vertebrates to become an outlier like the echinoderm. B) Salvador vertebrate topology matches the species tree. Among invertebrates the urochordate clusters tightly with vertebrates while the echinoderm and cephalochordate are outliers. The overall Salvador tree matches the species tree. The Bayesian tree (Figure S1B) shows one difference - the cephalochordate clusters outside the urochordate and vertebrate cluster with confidence.

**Figure 3:**
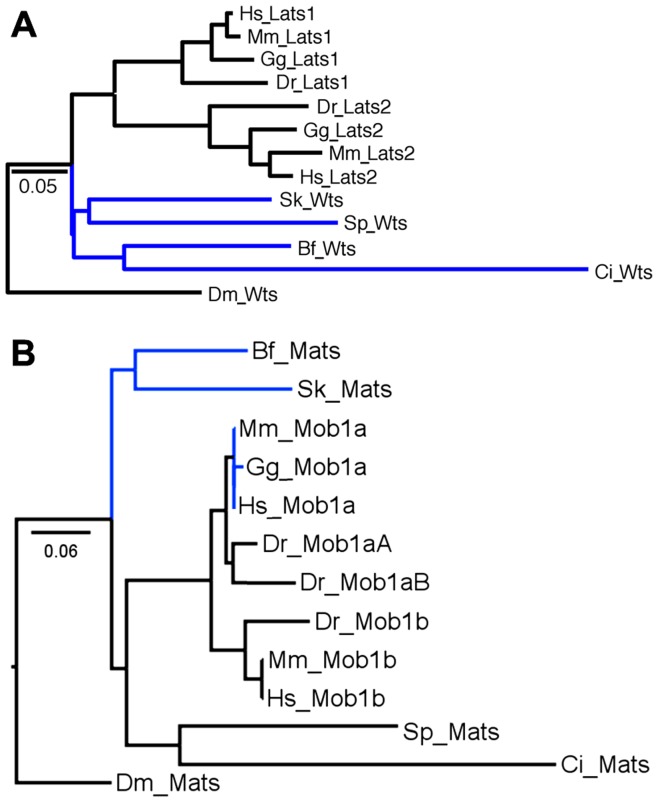
Warts/Lats and Mats/Mob Maximum Likelihood trees. A) Warts/Lats vertebrate topology matches the species tree. Among invertebrates, these proteins should be sequentially joined to the vertebrate cluster and thus this part of the Warts/Lats tree deviates from the species tree. The Bayesian tree (Figure S1C) shows one difference - rather than the hemichordate and echinoderm clustering together they are sequentially clustered with the large urochordate, cephalochordate and vertebrate group, an arrangement that better matches the species tree. B) Mats/Mob vertebrate topology matches the species tree except for difficulty resolving human, mouse and chicken Mob1a. Among invertebrates a cluster of the echinoderm and urochordate sequences is attached to the vertebrate cluster with the hemichordate and cephalochordates as outliers. The association of urochordates with vertebrates matches the species tree but the inclusion of echinoderms without hemichordates and cephalochordates does not. The Bayesian tree (Figure S1D) shows one difference - the urochordate switches places with the hemichordate leading to an arrangement that better matches the species tree but with no confidence.

**Figure 4:**
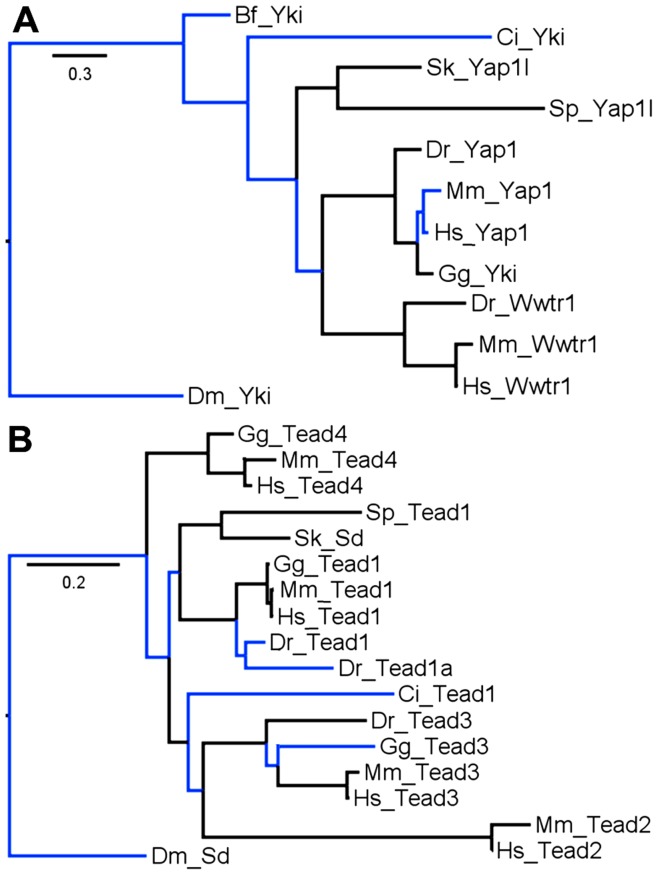
Yorkie/Yap/Taz and Scalloped/Tead Maximum Likelihood trees. A) Yorkie/Yap/Taz (Taz is formally known as Wwtr1) vertebrate topology matches the species tree. Among invertebrates there is strong pairing of the echinoderm and hemichordate but weak connections of this pair to cephalochordates, urochordates and vertebrates in relationships that deviate from the species tree. The Bayesian tree (Figure S1E) shows one difference - the urochordate has switched places with the cephalochordate exaggerating the deviation from the species tree. B) Scalloped/Tead vertebrate topology matches the species tree. Among invertebrates, the tree clusters the hemichordate and echinoderm with vertebrate Tead1 without the urochordate and clusters the urochordate with vertebrate Tead3, both are deviations from the species tree. The Bayesian tree (Figure S1F) shows one difference - the echinoderm and hemichordate move away from vertebrate Tead1 as outliers, an arrangement that is a slightly better match to the species tree.

In the Hippo/Mst family (Figure 2A) there are two members in each amniote (mammals and chicken). This is typical due to a vertebrate-specific whole-genome duplication that occurred in the Ordovician era [31]. However, in fish only Mst2 is present which is surprising since teleost fish experienced another whole-genome duplication after their divergence from other vertebrates in the Silurian era [32]. Thus in fish there are normally four copies of each gene/protein, but in the Hippo/Mst family fish have lost three copies. In the Salvador family there is only one member in each vertebrate (Figure 2B). This indicates loss of one copy in each amniote and three from fish. In the Warts/Lats family (Figure 3A) amniotes have the expected number but fish have lost two copies. In the Mats/Mob family (Figure 3B) mammals have the expected number but chicken and fish have each lost one copy. In the Yorkie/Yap/Taz family (Figure 4A) mammals have the expected number, but chicken has lost one and fish have lost two copies.

**Figure 5:**
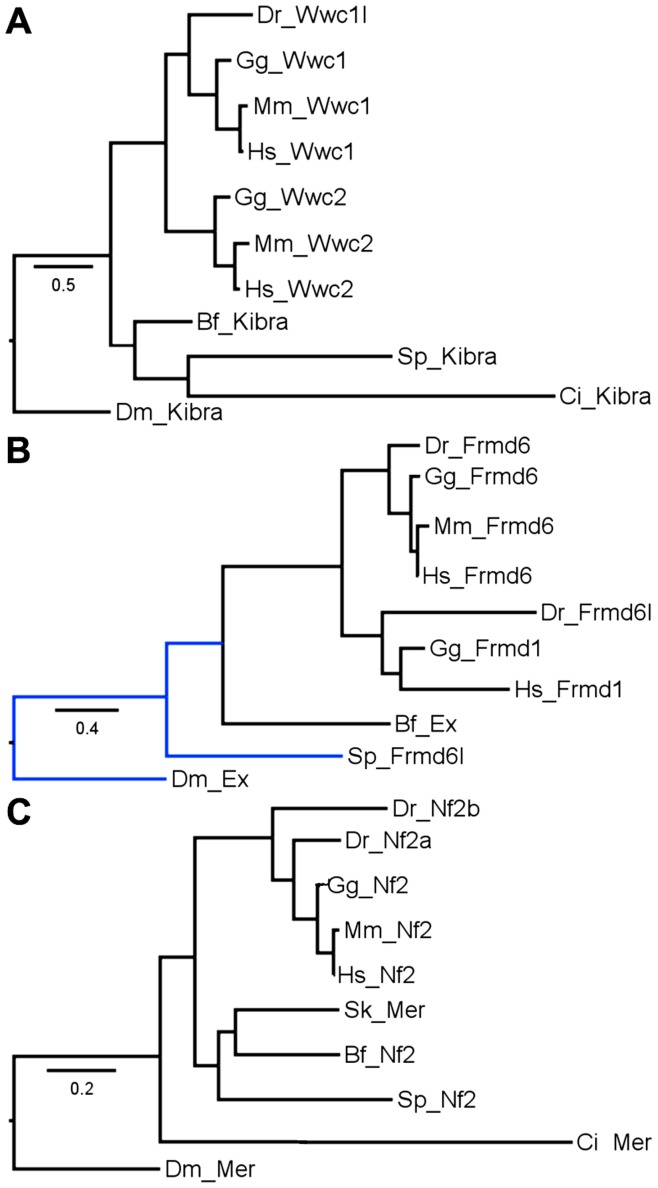
Kibra/Wwc, Expanded/Frmd and Merlin/Nf2 Maximum Likelihood trees. A) Kibra/Wwc vertebrate topology matches the species tree. Among invertebrates there is a significant cluster of the echinoderm and urochordate that excludes the cephalochordate, a deviation from the species tree. The Bayesian tree (Figure S1G) shows no differences. B) Expanded/Frmd vertebrate topology matches the species tree. *M. musculus* Frmd1 was found (NP_001191849.1) but excluded since it contains a mutation in the coding region that led it to be classified as a pseudogene. Our data showing this protein is well conserved in humans and chicken suggests that the mutation is a sequence error or belongs to a mutant allele rather than a pseudogene. Among invertebrates the cephalochordate strongly clusters with vertebrates and the echinoderm is an outlier matching the species tree. The Bayesian tree (Figure S1H) shows no differences. C) Merlin/Nf2 vertebrate topology matches the species tree. Among invertebrates, the hemichordate, cephalochordate and echinoderm form a strong cluster without the urochordate, an arrangement that deviates from the species tree. The Bayesian tree (Figure S1I) shows no differences.

The Scalloped/Tead family (Figure 4B) has a complex history. Each mammal has four members. Tead2 is unique to mammals as chicken and fish each have only three copies. Chicken has Tead1, 3 and 4 while fish have two Tead1s and Tead3. The mammal specific Tead2 cluster is statistically linked to the Tead3 cluster and both clusters form a group with Tead1. Both Tead3 and Tead1 are present in all vertebrates. The most parsimonious explanation for this distribution of Tead family members is that the ancestral vertebrate had four Tead genes as seen in mammals. Tead2 was lost in chicken and fish lost five Tead genes, a total of six events. Explaining the current distribution by ascribing two Tead genes to the ancestral vertebrate or by assigning Tead2 to a mammal-specific duplication requires a larger number of events. In the Kibra/WWc1 family (Figure 5A) amniotes have the expected number but fish have lost three copies. In the Expanded/Frmd family (Figure 5B) amniotes may have the expected number (see legend for a discussion of *M. musculus* Frmd1) but fish have lost two copies. In the Merlin/Nf2 family (Figure 5C) amniotes have each lost one copy while fish have lost two.

We then compared fly, mammal and fish copy number across the three pathways. As noted above, the expectation is two mammal and four fish genes/proteins for every one in flies. In mammalian TGF-β and Wnt, but not Hippo families, gene gain beyond this expectation is visible. For example: Smad1, 5, 8 are homologs of fly Mad [4] and Dvl1, 2, 3 are homologs of fly Dishevelled [33]. Alternatively, all pathways display gene loss in mammals. For Hippo, mammalian gene loss is seen in two of nine families (Salvador and Merlin/Nf2). For TGF-β (five families), mammalian gene loss is visible in the Co-Smad subfamily (Medea/Smad4). For Wnt (ten families), mammalian gene loss is visible in the β-catenin family.

Evidence of gene loss in Hippo families is also seen in fish. Hippo families in fish have universally experienced gene loss (down to a single copy in three families - Hippo/Mst, Salvador and Kibra/Wwc). Alternatively, many TGF-β and Wnt families in fish contain four or five copies reflecting both rounds of genome duplication. For example, Dishevelled is present in five copies and Smad2 is present in four copies [33],[34].

There is only one protein family in each of the TGF-β and Wnt pathways that is reduced to one copy in mammals and both of these proteins have pleiotropic functions. Smad4 belongs to the Co-Smad subfamily because it forms transcriptional complexes with many partners (Smads1, 2, 3, 5 and 8) and thus acts as a signal transducer for multiple TGF-β pathways. β-catenin family members play roles in Wnt signaling and in adherens junctions where they modulate the turnover of membrane-bound cadherins [10]. One possible explanation for copy number reduction in these multi-functional families is that this prevents unintended crosstalk between their various roles. Evolutionary mechanisms, such as adaptive mutations that prevent crosstalk are believed to strongly influence pathway diversification [35].

The logic that copy number reduction serves to prevent pathway crosstalk for Smad4 seems apt for Salvador. The sole copy of Salvador in vertebrates is thought to function as a co-factor for both Hippo/Mst kinases [11] just as the sole copy of Smad4 forms complexes with multiple Smad partners. Extending the similarity between these proteins to their roles as passive co-factors also seems reasonable. However, recently the view of Smad4 as a passive partner has been modified based on two reports: 1) that Smad4 monoubiquitylation cycles serves as highly efficient “off/on” switches for TGF-β signaling [8],[9] and 2) that the fly Co-Smad Medea can be shunted by cell type specific protein-protein interactions between Smad partners as an “inter-pathway” switch [36]. The similarities between Smad4 and Salvador in copy number and lysine conservation suggest at Salvador may function as an “off/on” switch for Hippo signaling via monoubiquitylation or as an “inter-pathway” switch between Mst1 and Mst2.

The logic that copy number reduction serves to prevent pathway crosstalk for Smad4 also seems apt for Merlin/Nf2. The sole copy of Merlin/Nf2 is thought to function primarily via protein-protein interactions that regulate the function of its many partners including Expanded/Frmd and Kibra/Wwc family members [29],[30]. Merlin/Nf2 is known to be regulated by phosphorylation and polyubiquitylation but the similarities between Smad4 and Merlin/Nf2 in copy number and lysine conservation suggest that Merlin/Nf2 may also be regulated via monoubiquitylation (as suggested by existing experimental data) or may play a role as an “inter-pathway” switch between Frmd1-Fermd6 or Wwc1-Wwc2 or Fermd and Wwc pathways.

### Salvador and Merlin/Nf2 Discordant Partner Substitution Rates are Similar to Smad4 Discordant Partner Substitution Rates

Unlike copy number and lysine conservation data that have small variable ranges, amino acid substitution rates in animals can vary up to 67,000-fold, For example, histoneH4 shows a rate of 0.001 substitutions per residue per billion years (all substitution rate data is stated using this standard metric and abbreviated as substitutions) while ATP-cone domain proteins show a rate of 66.6 substitutions [37],[38]. The latter rate is considered as fast as possible while still maintaining recognizable family membership.

First we calculated overall rates (Table 2) for six TGF-β families (range 0.258–1.339; average 0.861) and eleven Wnt families (range 0.333–1.730; average 1.184). We include the TGF-β and Wnt ligand families here, but not in the copy number analysis above since there is no *a priori* reason to expect a bias in substitution rates for ligands versus signal transducers whereas there is a well documented bias towards increasing copy number in ligands [39]. Overall, Hippo families are evolving more slowly than the other pathways (range 0.151–1.379; average 0.599).

**Table 2:** Overall amino acid substitution rates for families in the Wnt, Hippo and TGF-ß pathways.

Wnt Family	SubstitutionRate^a^	Hippo Family	SubstitutionRate	TGF-ß Family	Substitution Rate
Shaggy/Gsk3	**0.333**	Mats/Mob	0.151	R-Smad	**0.258**
Dsh	0.790	Hippo/Mst	**0.156**	Co-Smad	**0.595**
Wg/Wnt	0.861	Scalloped/Tead	0.291	TGF-ß-RI	0.830
Pangolin/TCF	1.021	Warts/Lats	0.298	I-Smad	0.990
Fz	1.024	Merlin/Nf2	0.498	TGF-ß-RII	1.153
APC	1.221	Kibra/Wwc	0.802	TGF-ß	1.339
Arm/ß-catenin	1.244	Salvador	**0.821**		
Arrow/LRP	1.505	Expanded/Frmd	0.997		
Axin	**1.583**	Yorkie/Yap/Taz	1.379		
Pygopus	1.712				
Legless/Bcl9	1.730				

a Substitution rate per residue per billion years shown in ascending order by pathway. For each pathway an example of two physically interacting proteins with concordant rates is underlined (e.g., Shaggy/Gsk3 and Axin) and an example of two physically interacting proteins with discordant rates is **bold** (e.g., Fz and Arrow/Lrp).

When examining interacting proteins with similar roles in the TGF-β and Wnt pathways we frequently saw comparable substitutions rates. Concordant rates suggest the proteins are under similar selective constraints. Examples of interacting proteins with concordant substitution rates are: 1) the TGF-β receptor complex components (Type I 0.830, Type II 1.153), 2) Wnt receptor complex components (Fz 1.024, LRP 1.505) and 3) Wnt transcription factor complexes (β-catenin 1.244, TCF 1.021). Alternatively interacting proteins in which each plays a distinct role frequently showed discordant rates (differing by more than 2-fold). An example in the TGF-β pathway is the Smad multimeric complex composed of Co-Smads regulated by monoubiquitylation (0.595) and receptor activated Smads regulated by phosphorylation (0.258). An example in the Wnt pathway is the multimeric complex composed of Shaggy/Gsk3 kinases (0.333) with the adapter proteins APC (1.221) and Axin (1.583).

In the Hippo pathway, we found examples of concordance such as: Warts/Lats kinase (0.298) and its co-factor Mats/Mob (0.151) as well as Kibra/Wwc (0.802) and Expanded/Frmd (0.997). Discordance in substitution rates between interacting proteins is evident for Hippo/Mst (0.156) and Salvador (.821; over five fold higher) as well as Merlin/Nf2 (0.498) and Expanded/Frmd (0.997) These findings are not expected from Salvador’s perceived role as a passive co-factor for the Hippo kinase or from the perceived roles of Merlin/Nf2 and Expanded/Frmd as simple adapter proteins. In summary, comparisons with discordant pairs in the TGF-β and Wnt pathways suggest that Salvador and Merlin/Nf2 have undiscovered roles distinct from their partners (e.g., monoubiquitylation), within the Hippo pathway or beyond.

Overall, this study builds upon successful phylogenetics-based predictions for the regulation of Smad signal transducers, including Smad4, in the TGF-β pathway. Comparison of phylogenetic data for nine Hippo families to results from studies of the TGF-β and Wnt pathways led us to predict new roles for Salvador and Merlin/Nf2 and to suggest that these may involve pathway regulation via monoubiquitylation. If validated, and suggestive experimental evidence is already visible in the literature for Merlin/Nf2, these predictions will reinforce the view that the creative exploitation of amino acid conservation via phylogenetics can illuminate new regulatory mechanisms affecting developmental pathways.

## Materials and Methods

### Sequences

All members of the Hippo/Mats, Salvador, Warts/Lats, Mats/Mob, Yorkie/Yap/Taz, Scalloped/Tead, Kibra/Wwc, Expanded/Frmd and Merlin/Nf2 families for nine fully sequenced organisms were retrieved and analyzed as described [10]. The longest isoform was utilized and partial sequences excluded. Sequences are named according to their genus and species. See Table S1 for accession numbers. Calculations of the overall amino acid substitution rate for each family were conducted in MEGA5 using the Poisson correction model [40].

### Alignments and Trees

Alignments for each gene family were created in MAFFT. In one case an alignment was annotated manually (in the Expanded/Frmd family an excessive tail on Expanded was removed). See Table S2 for detailed alignment length information. Maximum Likelihood trees (main text) were generated from alignments as described [10]. Bayesian trees (Figure S1) were generated from sequences aligned with MUSCLE [41] and created in MrBayes 3.1.1 [42]. For Bayesian trees, the prior amino acid model was set to Blosum. Gamma plus invariant sites were taken into account, with the gamma shape parameter being composed of four discrete categories. According to ProtTest [43], the gamma plus invariant model was the best-fit model for protein evolution in this family. The number of generations was set to 100, 000 with a sample frequency of 100 and burn-in frequency of 0.25. This was the smallest number of generations that produced meaningful trees. All other parameters were set to default values.

## Supporting Information

Figure S1 **Bayesian trees of Hippo pathway proteins.** Bayesian trees are displayed in the same format as the Maximum Likelihood trees in the main text with explicit posterior probabilities shown at the nodes. Branches with posterior probabilities <0.90 are considered weak and colored in blue. Branch lengths denote the number of amino acid changes per site. Organisms are abbreviated as in the Maximum Likelihood trees in the main text. A) Hippo/Mst Bayesian tree, B) Salvador Bayesian tree, C) Warts/Lats Bayesian tree, D) Mats/Mob Bayesian tree, E) Yorkie/Yap/Wwtr Bayesian tree, F) Scalloped/Tead Bayesian tree, G) Kibra/Wwc Bayesian tree, H) Expanded/Frmd Bayesian tree and I) Merlin/Nf2 Bayesian tree.
(PDF)

Table S1 **Accession numbers.**
(PDF)

Table S2 **Alignment lengths.** The total lengths of protein alignments used to create trees are shown below. Bayesian trees were made from MUSCLE alignments, while MAFFT alignments were used to create Maximum Likelihood trees. Expanded alignments were corrected by hand since the presence of numerous large *D. melanogaster* insertions and deletions resulted in many gaps, making it difficult to create trees. Original alignment lengths for Ex are shown in parentheses. Alignments are available upon request.
(PDF)
