# Supplementary material for: Hippo Pathway Phylogenetics Predicts Monoubiquitylation of Salvador and Merlin/Nf2
Source: PLoS One. 2012 Dec 14;7(12):e51599. doi: 10.1371/journal.pone.0051599 (PMC3522738; doi:10.1371/journal.pone.0051599)

Fig. S1A. Hippo/Mst Bayesian tree

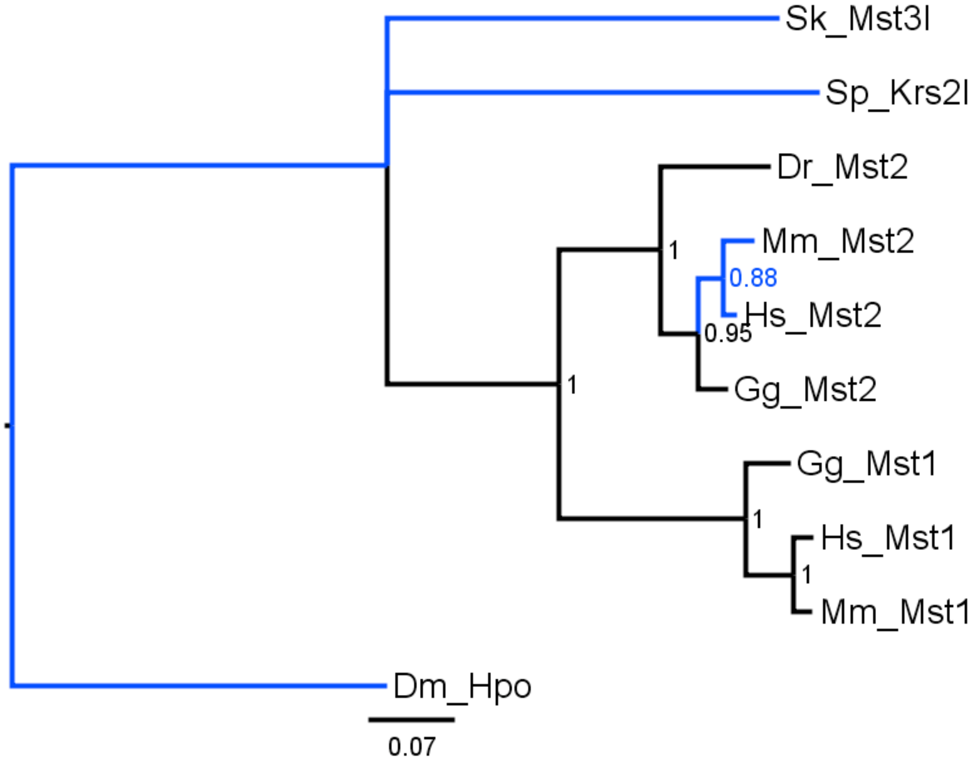

Fig. S1B. Salvador Bayesian tree

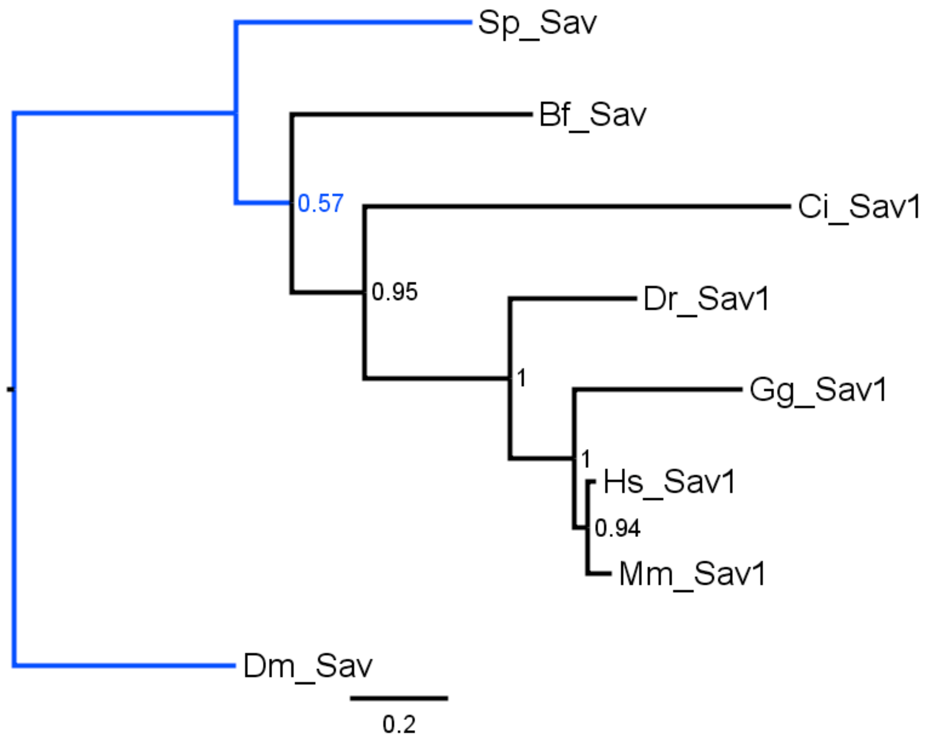

Fig. S1C. Warts/Lats Bayesian tree

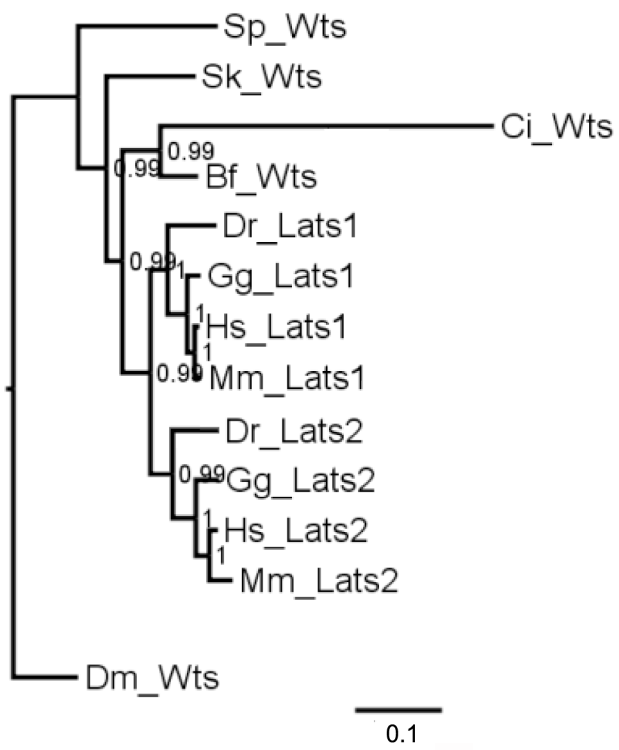

Fig. S1D. Mats/ Mob Bayesian tree

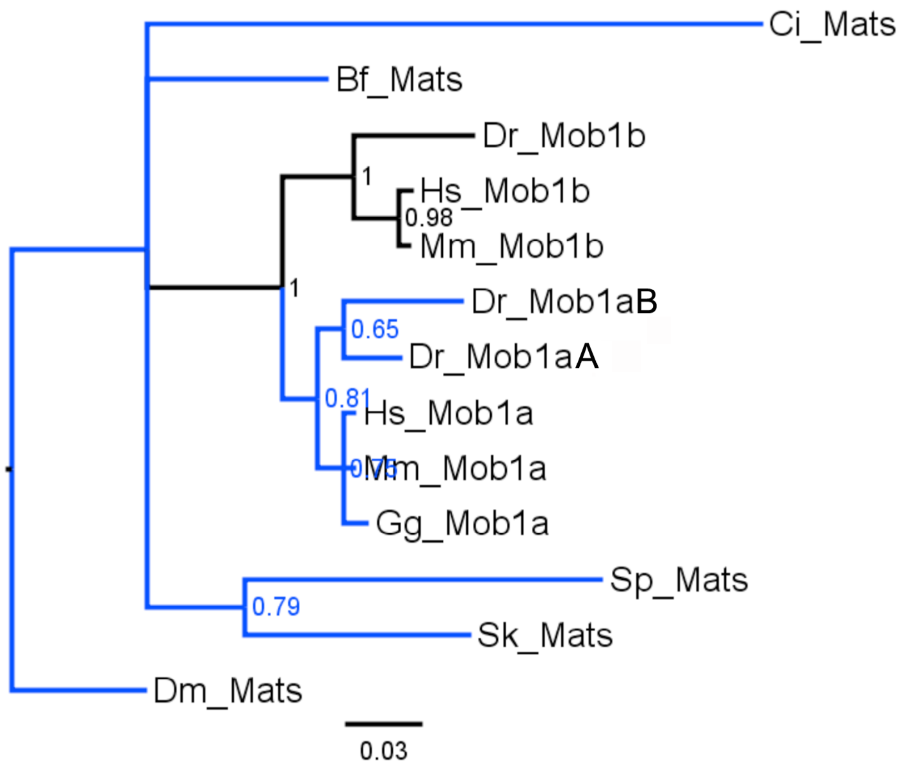

Fig. S1E. Yorkie/Yap/Wwtr Bayesian tree

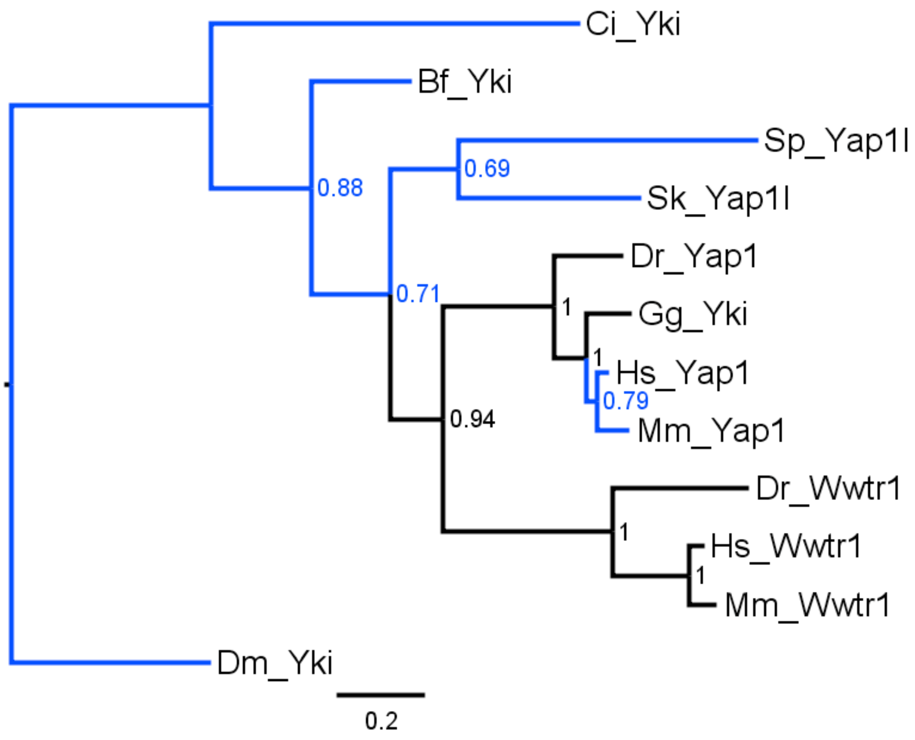

Fig. S1F. Scalloped/Tead Bayesian tree

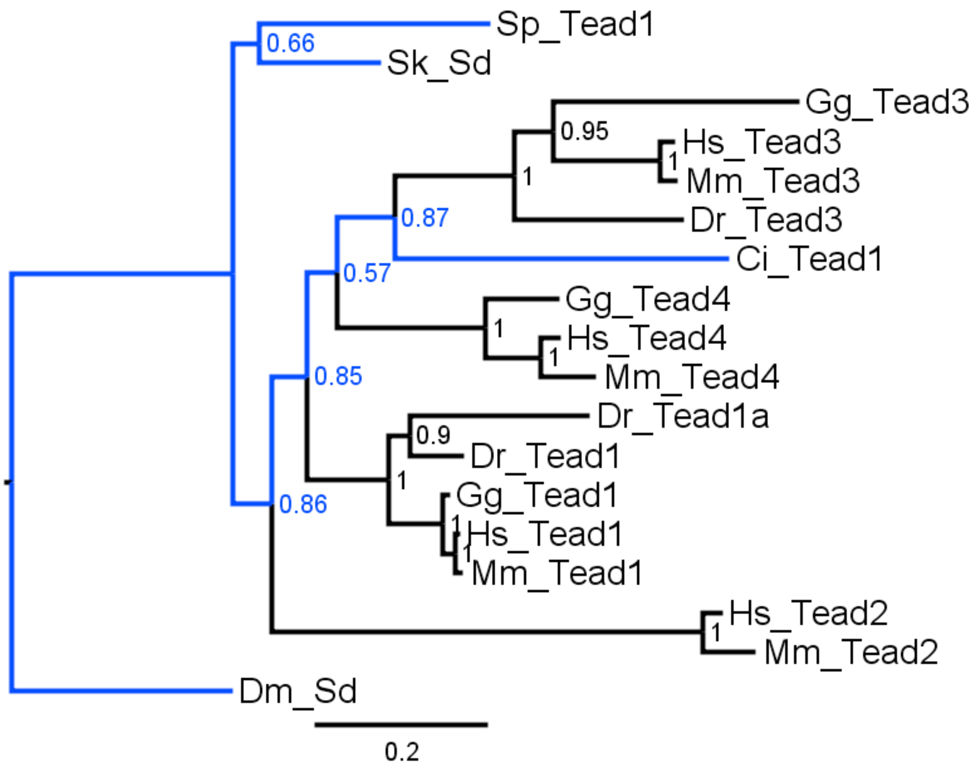

Fig. S1G. Kibra/Wwc Bayesian tree

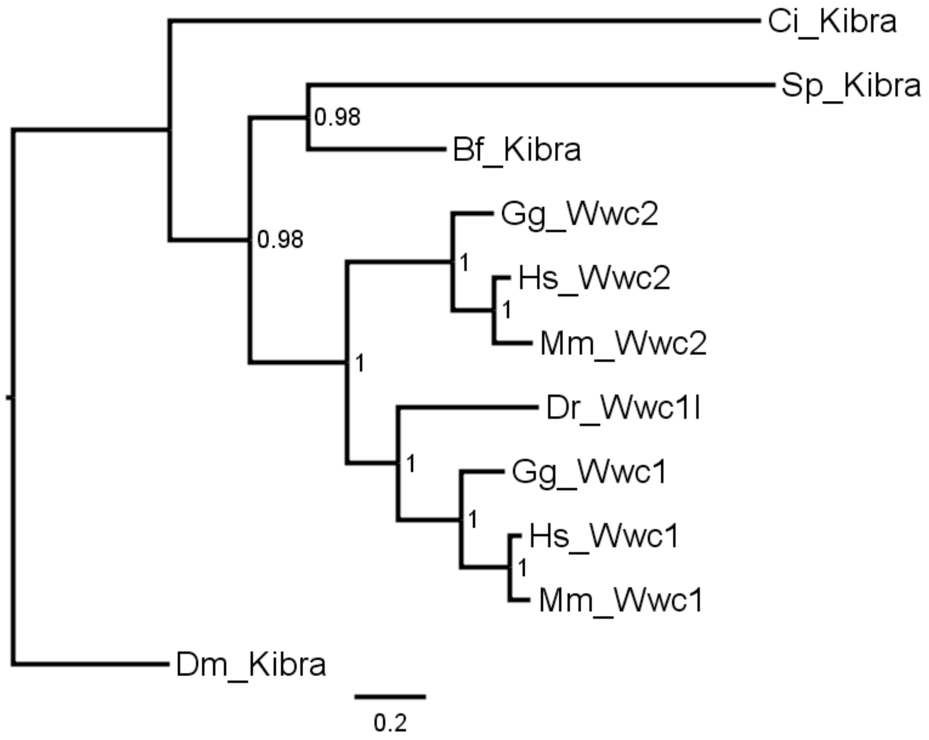

Fig. S1H. Expanded/Frmd Bayesian tree

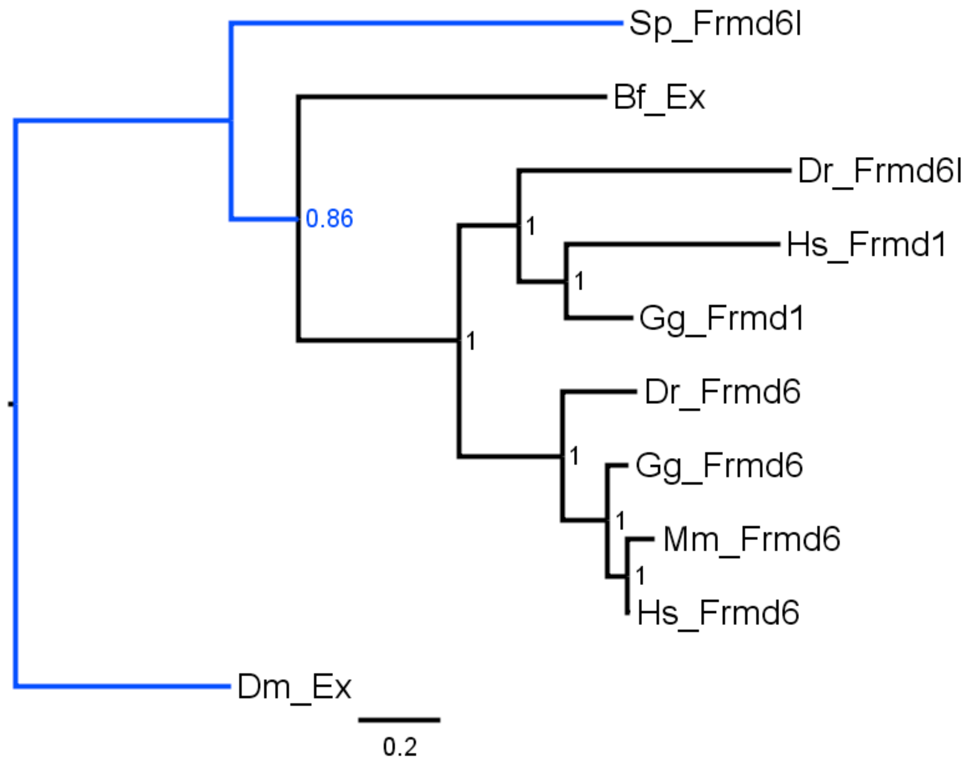

Fig. S1I. Merlin/Nf2 Bayesian tree

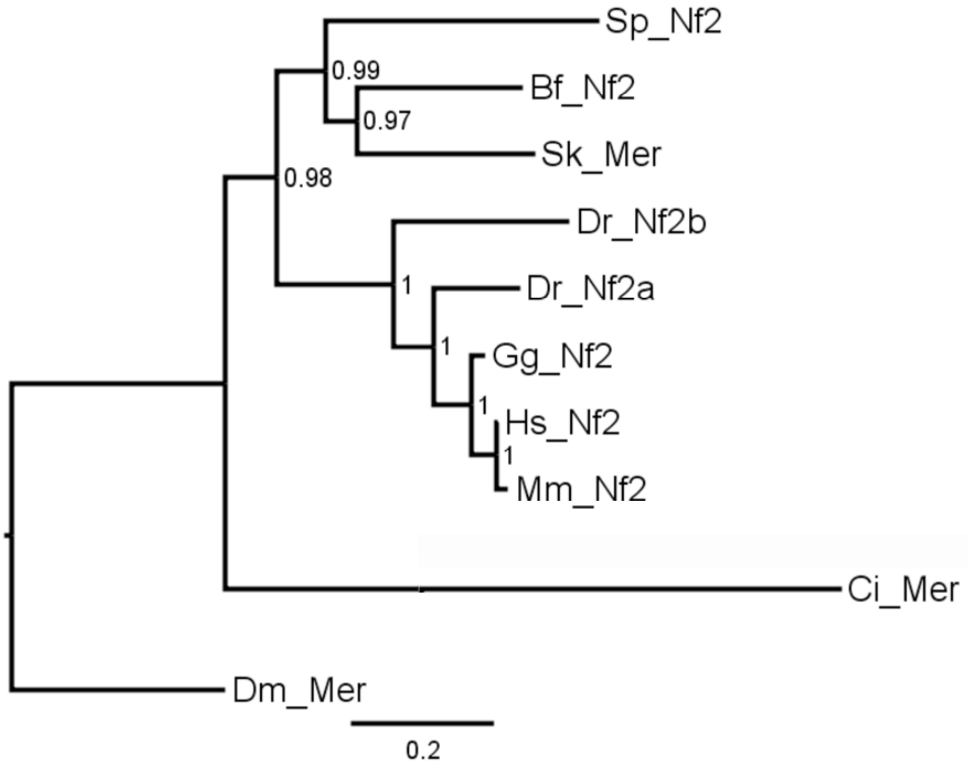

Supplement: Figure S1 — Bayesian trees of Hippo pathway proteins. Bayesian trees are displayed in the same format as the Maximum Likelihood trees in the main text with explicit posterior probabilities shown at the nodes. Branches with posterior probabilities <0.90 are considered weak and colored in blue. Branch lengths denote the number of amino acid changes per site. Organisms are abbreviated as in the Maximum Likelihood trees in the main text. A) Hippo/Mst Bayesian tree, B) Salvador Bayesian tree, C) Warts/Lats Bayesian tree, D) Mats/Mob Bayesian tree, E) Yorkie/Yap/Wwtr Bayesian tree, F) Scalloped/Tead Bayesian tree, G) Kibra/Wwc Bayesian tree, H) Expanded/Frmd Bayesian tree and I) Merlin/Nf2 Bayesian tree. (PDF) [file pone.0051599.s001.pdf]
