# Supplementary material for: Hippo Pathway Phylogenetics Predicts Monoubiquitylation of Salvador and Merlin/Nf2
Source: PLoS One. 2012 Dec 14;7(12):e51599. doi: 10.1371/journal.pone.0051599 (PMC3522738; doi:10.1371/journal.pone.0051599)
Supplement: Table S1 — Accession numbers. (PDF) [file pone.0051599.s002.pdf]

Supplemental information

Table S1. Accession numbers

Hippo/Mst family: Dm_Hpo, NP_611427.1; Hs_Mst1, NP_006273.1; Hs_Mst2, NP_006272.2; Mm_Mst1, NP_067395.1; Mm_Mst2, NP_062609.2; Gg_Mst1, NP_001026024.1; Gg_Mst2, NP_001026508.1; Dr_Mst2, NP_955966.1; Sk_Mst3l, XP_002736706.1 and Sp Krs2l, XP_781787.2. A partial sequence for *B. floridae* was found (XP_002589064.1) but excluded. Reciprocal Blasting revealed *D. rerio* Mst2 was also the best hit when searching with Mst1. Additional *D. rerio* hits with Mst1 or Mst2 were more closely related to other serine-threonine kinases. No strong hits were found for *C. intestinalis*.

Salvador family: Dm_Sav, NP_788721.1; Hs_Sav1, NP_068590.1; Mm_Sav1, NP_071311.1; Gg_Sav1, XP_421467.2; Dr_Sav1, NP_001004560.1; Bf_Sav, XP_002610502.1; Ci_Sav1, XP_002126709.1 and Sp_Sav XP_001178982.1. No strong hits were found for *S. kowalevskii*.

Warts/Lats family: Dm_Wts, NP_733403.1; Hs_Lats1, NP_004681.1; Hs_Lats2, NP_055387.2; Mm_Lats1, NP_034820.1; Mm_Lats2, NP_056586.2; Gg_Lats1, XP_419666.2; Gg_Lats2, XP_417143.2; Dr_Lats1, NP_001018346.1; Dr_Lats2, NP_001121728.1; Bf_Wts, XP_002587173.1; Ci_Wts, XP_002124396.1; Sk_Wts, XP_002738496.1 and Sp_Wts, XP_795100.2.

Mats/Mob family: Dm_Mats, NP_651041.3; Hs_Mob1a, NP_775739.1; Hs_Mob1b, NP_060691.2; Mm_Mob1a, NP_081011.1; Mm_Mob1b, NP_663546.1; Gg_Mob1a, XP_420601.1; Dr_Mob1aa, NP_956494.1; Dr_Mob1ab, NP_999948.2; Dr_Mob1b, NP_956208.1; Bf_Mats, XP_002596178.1; Ci_Mats, XP_002122354.1; Sk_Mats, XP_002741421.1 and Sp_Mats, XP_788775.1.

Yorkie/Yap/Wwtr family: Dm_Yki, NP_001036568.2; Hs_Yap1, NP_001123617.1; Hs_Wwtr1, NP_056287.1; Mm_Yap1, NP_033560.1; Mm_Wwtr1, NP_598545.2; Gg_Yki, NP_990574.1; Dr_Yap1, NP_001132952.1; Dr_Wwtr1, NP_001032785.1; Bf_Yki, XP_002595229.1; Ci_Yki, XP_002130260.1; Sk_Yap1l, XP_002734168.1 and Sp_Yap1l,

XP_789542.2. Note that Wwtr1 is also referred to as TAZ.

Scalloped/Tead family: Dm_Sd, NP_511169.1; Hs_Tead1, NP_068780.2; Hs_Tead2, NP_003589.1; Hs_Tead3, NP_003205.2; Hs_Tead4, NP_003204.2; Mm_Tead1, NP_033372.1; Mm_Tead2, NP_035695.1; Mm_Tead3, NP_001091696.2; Mm_Tead4, NP_035697.3; Gg_Tead1, XP_420962.2; Gg_Tead3, XP_419267.1; Gg_Tead4, NP_001001339.1; Dr_Tead1, XP_001345193.2; Dr_Tead1a, NP_998012.1; Dr_Tead3, NP_001103194.1; Ci_Tead1, NP_001087209.1; Sk_Sd, NP_001161650.1 and Sp_Tead1, XP_785374.2. A partial sequence for *B. floridae* was also found (XP_002595807.1), but was excluded.

Kibra/Ww family: Dm_Kibra, NP_001034055.1; Hs_Wwc1, NP_056053.1; Hs_Wwc2, NP_079225.5; Mm_Wwc1, NP_740749.1; Mm_Wwc2, NP_598552.2; Gg_Wwc1, XP_414499.2; Gg_Wwc2, XP_420516.2; Dr_Wwc1l, XP_689275.3; Bf_Kibra, XP_002598149.1; Ci_Kibra, XP_002129513.1 and Sp_Kibra, XP_780710.2. Dm Kibra is also called CG33967. Some *D. rerio* sequences with high similarity via reciprocal blasting were discontinued in GenBank and thus excluded. No strong hits were found for *S. kowalevskii*.

Expanded/Fermd family: Dm_Ex, NP_476840.2; Hs_Frmd1, NP_079195.3; Hs_Frmd6, NP_001035946.1; Mm_Frmd6, NP_082403.2; Gg_Frmd1, XP_419603.2; Gg_Frmd6, XP_426484.2; Dr_Frmd6, XP_002664244.1; Dr_Frmd6l, NP_001104657.1; Bf_Ex, XP_002590857.1 and Sp_Frmd6l, XP_797238.2. A sequence for *M. musculus* Frmd1 is present, but was excluded because it appeared to have a translation discrepancy (LOC544719). No strong hits were found in *S. kowalevskii*, though many other ezrin/radixin/moesin members are clearly present. Two potential tandem *C. intestinalis* sequences (XP_002125027.1 and XP_002125095.1) were excluded as we took a conservative approach and only analyzed sequences with significant e-value matches to the query sequence.

Merlin/Nf2 family: Dm_Mer, NP_523413.1; Hs_Nf2, NP_000259.1; Mm_Nf2, NP_035028.2; Gg_Nf2, NP_989828.2; Dr_Nf2a, NP_001122179.1; Dr_Nf2b, NP_998116.1; Bf_Nf2, XP_002591890.1; Ci_Mer, XP_002125990.1; Mt_Mer, BAF49216.1; Sk_Mer, NP_001164711.1 and Sp_Nf2, XP_781142.2.
