# Supplementary material for: Hippo Pathway Phylogenetics Predicts Monoubiquitylation of Salvador and Merlin/Nf2
Source: PLoS One. 2012 Dec 14;7(12):e51599. doi: 10.1371/journal.pone.0051599 (PMC3522738; doi:10.1371/journal.pone.0051599)
Supplement: Table S2 — Alignment lengths. The total lengths of protein alignments used to create trees are shown below. Bayesian trees were made from MUSCLE alignments, while MAFFT alignments were used to create Maximum Likelihood trees. Expanded alignments were corrected by hand since the presence of numerous large D. melanogaster insertions and deletions resulted in many gaps, making it difficult to create trees. Original alignment lengths for Ex are shown in parentheses. Alignments are available upon request. (PDF) [file pone.0051599.s003.pdf]

Table S2. Alignment lengths. The total lengths of protein alignments used to create trees are shown below. Bayesian trees were made from MUSCLE alignments, while MAFFT alignments were used to create Maximum Likelihood trees. Expanded alignments were corrected by hand as many long *D. melanogaster* indels resulted in a large number of gaps, making it difficult to create trees. Original alignment lengths for Ex are shown in parentheses. Alignments are available upon request.

Protein	MUSCLE length	MAFFT length
Hippo (Hpo)	676	682
Salvador (Sav)	763	737
Warts (Wts)	2637	3366
Mats (Mats)	227	227
Yorkie (Yki)	732	787
Scalloped (Sd)	583	639
Kibra	1526	1556
Expanded (Ex)	648 (1495)	648 (1536)
Merlin (Mer)	737	739
